# Graph Neural Networks for Maximum Constraint Satisfaction

**DOI:** 10.3389/frai.2020.580607

**Published:** 2021-02-25

**Authors:** Jan Tönshoff, Martin Ritzert, Hinrikus Wolf, Martin Grohe

**Affiliations:** Chair of Computer Science 7 (Logic and Theory of Discrete Systems), Department of Computer Science, RWTH Aachen University, Aachen, Germany

**Keywords:** graph neural networks, combinatorial optimization, unsupervised learning, constraint satisfaction problem, graph problems, constraint maximization

## Abstract

Many combinatorial optimization problems can be phrased in the language of constraint satisfaction problems. We introduce a graph neural network architecture for solving such optimization problems. The architecture is generic; it works for all binary constraint satisfaction problems. Training is unsupervised, and it is sufficient to train on relatively small instances; the resulting networks perform well on much larger instances (at least 10-times larger). We experimentally evaluate our approach for a variety of problems, including Maximum Cut and Maximum Independent Set. Despite being generic, we show that our approach matches or surpasses most greedy and semi-definite programming based algorithms and sometimes even outperforms state-of-the-art heuristics for the specific problems.

## 1 Introduction

Constraint satisfaction is a general framework for casting combinatorial search and optimization problems; many well-known NP-complete problems, for example, *k*-colorability, Boolean satisfiability and maximum cut can be modeled as constraint satisfaction problems (CSPs). Our focus is on the optimization version of constraint satisfaction, usually referred to as maximum constraint satisfaction (Max-CSP), where the objective is to satisfy as many constraints of a given instance as possible. There is a long tradition of designing exact and heuristic algorithms for all kinds of CSPs. Our work should be seen in the context of a recently renewed interest in heuristics for NP-hard combinatorial problems based on neural networks, mostly GNNs (for example, Khalil et al., 2017; Selsam et al., 2019; Lemos et al., 2019; Prates et al., 2019).

We present a generic graph neural network (GNN) based architecture called RUN-CSP (Recurrent Unsupervised Neural Network for Constraint Satisfaction Problems) with the following key features:

**Unsupervised:** Training is unsupervised and just requires a set of instances of the problem.

**Scalable:** Networks trained on small instances achieve good results on much larger inputs.

**Generic:** The architecture is generic and can learn to find approximate solutions for any binary Max-CSP.

We remark that in principle, every CSP can be transformed into an equivalent binary CSP (see Section 2 for a discussion).

To solve Max-CSPs, we train a GNN, which we view as a message passing protocol. The protocol is executed on a graph with nodes for all variables and edges for all constraints of the instance. After running the protocol for a fixed number of rounds, we extract probabilities for the possible values of each variable from its current state. All parameters determining the messages, the update of the internal states, and the readout function are learned. Since these parameters are shared over all variables, we can apply the model to instances of arbitrary size^1^. Our loss function rewards solutions with many satisfied constraints. Thus, our networks learn to satisfy the maximum number of constraints which naturally puts the focus on the optimization version Max-CSP of the constraint satisfaction problem.

This focus on the optimization problem allows us to train unsupervised, which is a major point of distinction between our work and recent neural approaches to Boolean satisfiability (Selsam et al., 2019) and the coloring problem (Lemos et al., 2019). Both approaches require supervised training and output a prediction for satisfiability or coloring number. Furthermore, our approach not only returns a prediction whether the input instance is satisfiable, but it returns an (approximately optimal) variable assignment. The variable assignment is directly produced by a neural network, which distinguishes our end-to-end approach from methods that combine neural networks with conventional heuristics, such as Khalil et al., (2017) and Li et al., (2018).

**FIGURE 1:**
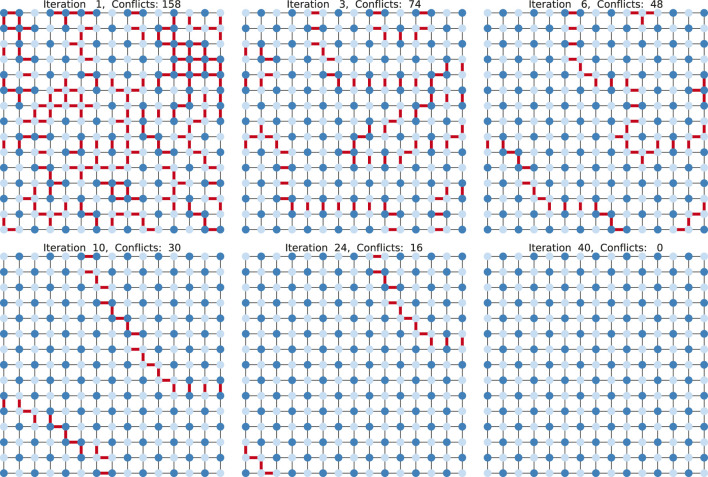
A 2-coloring for a grid graph found by RUN-CSP in 40 iterations. Conflicting edges are shown in red.

We experimentally evaluate our approach on the following NP-hard problems: the maximum 2-satisfiability problem (Max-2-SAT), which asks for an assignment maximizing the number of satisfied clauses for a given Boolean formula in 2-conjunctive normal form; the maximum cut problem (Max-Cut), which asks for a partition of a graph in two parts such that the number of edges between the parts is maximal (see Figure 1); the 3-colorability problem (3-COL), which asks for a 3-coloring of the vertices of a given graph such that the two endvertices of each edge have distinct colors. We also consider the maximum independent set problem (Max-IS), which asks for an independent set of maximum cardinality in a given graph. Strictly speaking, Max-IS is not a maximum constraint satisfaction problem, because its objective is not to maximize the number of satisfied constraints, but to satisfy all constraints while maximizing the number of variables with a certain value. We include this problem to demonstrate that our approach can easily be adapted to such related problems.

Our experiments show that our approach works well for all four problems and matches competitive baselines. Since our approach is generic for all Max-CSPs, those baselines include other general approaches such as greedy algorithms and semi-definite programming (SDP). The latter is particularly relevant, because it is known (under certain complexity theoretic assumptions) that SDP achieves optimal approximation ratios for all Max-CSPs (Raghavendra, 2008). For Max-2-SAT, our approach even manages to surpass a state-of-the-art heuristic. In general, our method is not competitive with the highly specialized state-of-the-art heuristics. However, we demonstrate that our approach clearly improves on the state-of-the-art for neural methods on small and medium-sized binary CSP instances, while still being completely generic. We remark that our approach does not give any guarantees, as opposed to some traditional solvers which guarantee that no better solution exists.

Almost all models are trained on quite small training sets consisting of small random instances. We evaluate those models on unstructured random instances as well as more structured benchmark instances. Instance sizes vary from small instances with 100 variables and 200 constraints to medium sized instances with more than 1,000 variables and over 10,000 constraints. We observe that RUN-CSP is able to generalize well from small instances to instances both smaller and much larger. The largest (benchmark) instance we evaluate on has approximately 120,000 constraints, but that instance required the use of large training graphs. Computations with RUN-CSP are very fast in comparison to many heuristics and profit from modern hardware like GPUs. For medium-sized instances with 10,000 constraints inference takes less than 5 s.

### 1.1 Related Work

Traditional methods for solving CSPs include combinatorial constraint propagation algorithms, logic programming techniques and domain specific approaches, for an overview see Apt (2003), Dechter (2003). Our experimental baselines include a wide range of classical algorithms, mostly designed for specific problems. For Max-2-SAT, we compare the performance to that of *WalkSAT* (Selman et al., 1993; Kautz, 2019), which is a popular stochastic local search heuristic for Max-SAT. Furthermore, we use the state-of-the-art Max-SAT solver *Loandra* (Berg et al., 2019), which combines linear search and core-guided algorithms. On the Max-Cut problem, we compare our method to multiple implementations of a heuristic approach by Goemans and Williamson (1995). This method is based on semi-definite programming (SDP) and is particularly popular since it has a proven approximation ratio of α≃0.878. Other Max-Cut baselines utilize extremal optimization (Boettcher and Percus, 2001) and local search (Benlic and Hao, 2013). For Max-3-Col, we measure the results against *HybridEA* (Galinier and Hao, 1999; Lewis et al., 2012; Lewis, 2015), which is an evolutionary algorithm with state-of-the-art performance. Furthermore, a simple greedy coloring heuristic (Brélaz, 1979) is also used as a comparison. *ReduMIS* is a state-of-the-art Max-IS solver that combines kernelization techniques and evolutionary algorithms. We use it as a Max-IS baseline, together with a simple greedy algorithm.

Beyond these traditional approaches there have been several attempts to apply neural networks to NP-hard problems and more specifically CSPs. An early group of papers dates back to the 1980s and uses Hopfield Networks (Hopfield and Tank, 1985) to approximate TSP and other discrete problems using neural networks. Hopfield and Tank use a single-layer neural network with sigmoid activation and apply gradient descent to come up with an approximative solution. The loss function adopts soft assignments and uses the length of the TSP tour and a term penalizing incorrect tours as loss, hence being unsupervised. This approach has been extended to *k*-colorability (Dahl, 1987; Takefuji and Lee, 1991; Gassen and Carothers, 1993; Harmanani et al., 2010) and other CSPs (Adorf and Johnston, 1990). The loss functions used in some of these approaches are similar to ours.

Newer approaches involve modern machine learning techniques and are usually based on GNNs. NeuroSAT (Selsam et al., 2019), a learned message passing network for predicting satisfiability, reignited the interest in solving NP-complete problems with neural networks. Prates et al., (2019) use GNNs to learn TSP and trained on instances of the form (G,ℓ±ε) where ℓ is the length of an optimal tour on *G*. They achieved good results on graphs with up to 40 nodes. Using the same idea, Lemos et al., (2019) learned to predict *k*-colorability of graphs scaling to larger graphs and chromatic numbers than seen during training. Yao et al., (2019) evaluated the performance of unsupervised GNNs for the Max-Cut problem. They adapted a GNN architecture by Chen et al., (2019) to Max-Cut and trained two versions of their network, one through policy gradient descent and the other via a differentiable relaxation of the loss function which both achieved similar results. Amizadeh et al., (2019) proposed an unsupervised architecture for Circuit-SAT, which predicts satisfying variable assignments for a given formula. Khalil et al., (2017) proposed an approach for combinatorial graph problems that combines reinforcement learning and greedy search. They iteratively construct solutions by greedily adding nodes according to estimated scores. The scores are computed by a neural network, which is trained through Q-Learning. They test their method on the MVC, Max-Cut, and TSP problems, where they outperform traditional heuristics across several benchmark instances. For the #P-hard weighted model counting problem for DNF formulas, Abboud et al., (2019) applied a GNN-based message passing approach. Finally, Li et al., (2018) use a GNN to guide a tree search for Max-IS.

## 2 Constraint Satisfaction Problems

Formally, a *CSP-instance* is a triple I=(X,D,C), where *X* is a set of variables, *D* is a domain, and *C* is a set of constraints of the form $\left(x_{1},\ldots ,x_{\ell },R\right)$ for some $R\subseteq D^{\ell }$. A *constraint language* is a finite set Γ of relations over some fixed domain *D*, and *I* is a Γ-instance if R∈Γ for all constraints $\left(x_{1},\ldots ,x_{\ell },R\right)\in C$. An *assignment* α:X→D satisfies a constraint $\left(x_{1},\ldots ,x_{\ell },R\right)$ if $\left(\alpha \left(x_{1}\right),\ldots ,\alpha \left(x_{\ell }\right)\right)\in R$, and it satisfies the instance *I* if it satisfies all constraints in *C*. CSP(Γ) is the problem of deciding whether a given Γ-instance has a satisfying assignment and finding such an assignment if there is one. Max −CSP(Γ) is the problem of finding an assignment that satisfies the maximum number of constraints.

For example, an instance of 3-COL has a variable $x_{v}$ for each vertex *v* of the input graph, domain D={1,2,3}, and a constraint $\left(v,w,R_{\neq }^{3}\right)$ for each edge vw of the graph. Here, $R_{\neq }^{3}=\left\{\left(1,2\right),\left(2,1\right),\left(1,3\right),\left(3,1\right),\left(2,3\right),\left(3,2\right)\right\}$ is the inequality relation on {1,2,3}. Thus 3-COL is $\text{CSP}\left(\left\{R_{\neq }^{3}\right\}\right)$.

In this paper, we only consider *binary CSPs*, that is, CSPs whose constraint language only contains unary and binary relations. From a theoretical perspective, this is no real restriction, because it is well known that every CSP can be transformed into an “equivalent” binary CSP (see Dechter, 2003). Let us review the construction. Suppose we have a constraint language Γ of maximum arity k≥3 over some domain *D*. We construct a binary constraint language $\hat{\text{Γ}}$ as follows. The domain $\hat{D}$ of $\hat{\text{Γ}}$ consists of all elements of *D* as well as all pairs (a,R) where R∈Γ and a is a tuple occurring in *R*. For every R∈Γ, we add a unary relation $Q_{R}$ consisting of all pairs $\left(a,R\right)\in \hat{D}$ where a∈R. Moreover, for 1≤i≤k we add a binary “projection” relation $P_{i}$ consisting of all pairs $\left(\left(a,R\right),a_{i}\right)$ for R∈Γ, say of arity ℓ≤k, and $a=\left(a_{1},\ldots ,a_{\ell }\right)\in R$. Finally, for every instance I=(X,D,C) of CSP(Γ) we construct an instance $\hat{I}=\left(\hat{X},\hat{D},\hat{C}\right)$ of $\text{CSP}\left(\hat{\text{Γ}}\right)$, where $\hat{X}$ consists of all variables in *X* and a new variable $y_{c}$ for every constraint $c=\left(x_{1},\ldots ,x_{\ell },R\right)\in C$ and $\hat{C}$ consists of a *tuple constraint* $\left(y_{c},Q_{R}\right)$ and *projection constraints* $\left(y_{c},x_{i},P_{i}\right)$ for all 1≤i≤ℓ≤k. Here, the tuple constraints select for every constraint $c=\left(\bar{x},R\right)\in C$ a tuple a∈R and the projection constraints ensure a consistent assignment to the original variables $X\subseteq \hat{X}$. Then the instances *I* and $\hat{I}$ are equivalent in the sense that *I* is satisfiable if and only if $\hat{I}$ is and there is a one-to-one correspondence between the satisfying assignments.

However, the construction is not approximation preserving. For example, it is not the case that an assignment satisfying 90% of the constraints of $\hat{I}$ yields an assignment satisfying 90% of the constraints of *I*. It is possible to fix this by adding weights to the constraints, making it more expensive to violate projection constraints. Moreover, and arguably more importantly in this context, it is not clear how well our method works on CSPs of higher arity when translated to binary CSPs using this construction. We leave a thorough experimental evaluation of CSPs with higher arities for future work.

## 3 Method

### 3.1 Architecture

We use a randomized recurrent GNN architecture to evaluate a given problem instance using message passing. For any binary constraint language Γ a RUN-CSP network can be trained to approximate Max ‐CSP(Γ). Intuitively, our network can be viewed as a trainable communication protocol through which the variables of a given instance can negotiate a value assignment. With every variable x∈X we associate a short-term state $s_{x}^{\left(t\right)}\in {\mathbb{R}}^{k}$ and a hidden (long-term) state $h_{x}^{\left(t\right)}\in {\mathbb{R}}^{k}$ which change throughout the message passing iterations $t\in \left\{0,\ldots ,t_{\text{max}}\right\}$. The short-term state vector $s_{x}^{\left(0\right)}$ for every variable *x* is initialized by sampling each value independently from a normal distribution with zero mean and unit variance. All hidden states $h_{x}^{\left(0\right)}$ are initialized as zero vectors.

Every message passing step uses the same weights and thus we are free to choose the number $t_{\text{max}}\in \mathbb{N}$ of iterations for which RUN-CSP runs on a given problem instance. This number may or may not be identical to the number of iterations used for training. The state size *k* and the number of iterations used for training $t_{\text{max}}^{\text{tr}}$ and evaluation $t_{\text{max}}^{\text{ev}}$ are the main hyperparameters of our network.

Variables *x* and *y* that co-occur in a constraint c=(x,y,R) can exchange messages. Each message depends on the states $s_{x}^{\left(t\right)},s_{y}^{\left(t\right)}$, the relation *R*, and the order of *x* and *y* in the constraint but not on the internal long-term states $h_{x}^{\left(t\right)},h_{y}^{\left(t\right)}$. The dependence on *R* implies that we have independent message generation functions for every relation *R* in the constraint language Γ. The process of message passing and updating the internal states is repeated $t_{\text{max}}$ times. We use linear functions to compute the messages as preliminary experiments showed that more complicated functions did not improve performance while being less stable and less efficient during training. Thus, the messaging function for every relation *R* is defined by a trainable weight matrix $M_{R}\in {\mathbb{R}}^{2k\times 2k}$ as

$$S_{R}\left(s_{x}^{\left(t\right)},s_{y}^{\left(t\right)}\right)=M_{R}\left(\begin{array}{c} s_{x}^{\left(t\right)}\\ s_{y}^{\left(t\right)} \end{array}\right).$$ (1)

The output of $S_{R}$ consists of two stacked *k*-dimensional vectors, which represent the messages to *x* and *y*, respectively. Note that the generated messages depend on the order of the variables in the constraint. This behavior is desirable for asymmetric relations. For symmetric relations we modify $S_{R}$ to produce messages independently from the order of variables in *c*. In this case we use a smaller weight matrix $M_{R}\in {\mathbb{R}}^{k\times 2k}$ to generate both messages. Note that the two messages can still be different, but the content of each message depends only on the states of the endpoints.

The internal states $h_{x}$ and $s_{x}$ are updated by an LSTM cell based on the mean of the received messages. For a variable *x* which received the messages $m_{1},\ldots ,m_{\ell }$ the new states are thus computed by

$$h_{x}^{\left(t+1\right)},s_{x}^{\left(t+1\right)}=\text{LSTM}\left(h_{x}^{\left(t\right)},\;s_{x}^{\left(t\right)},\;\frac{1}{\ell }\overset{\ell }{\underset{i=1}{\sum }}m_{i}\right).$$ (2)

For every variable *x* and iteration $t\in \left\{1,\ldots ,t_{\text{max}}\right\}$, the network produces a soft assignment $\varphi^{\left(t\right)}\left(x\right)$ from the state $s_{x}^{\left(t\right)}$. In our architecture we use $\varphi^{\left(t\right)}\left(x\right)=\text{softmax}\left(Ws_{x}^{\left(t\right)}\right)$ with $W\in {\mathbb{R}}^{d\times k}$ trainable and d=|D| (domain size of the CSP). In *φ*, the linear function reduces the dimensionality while the softmax function enforces stochasticity. The soft assignments $\varphi^{\left(t\right)}\left(x\right)$ can be interpreted as probabilities of a variable *x* receiving a certain value v∈D. If the domain *D* contains only two values, we compute a “probability” $p^{\left(t\right)}\left(x\right)=\sigma \left(Ws_{x}^{\left(t\right)}\right)$ for each node with $W\in {\mathbb{R}}^{1\times k}$. The soft assignment is then given by $\varphi^{\left(t\right)}\left(x\right)=\left(p^{\left(t\right)}\left(x\right),1-p^{\left(t\right)}\left(x\right)\right)$. To obtain a hard variable assignment $\alpha^{\left(t\right)}:X\to D$, we assign the value with the highest estimated probability in $\varphi^{\left(t\right)}\left(x\right)$ for each variable x∈X. From the hard assignments $\alpha^{\left(1\right)},\ldots ,\alpha^{\left(t_{\text{max}}^{\text{ev}}\right)}$, we select the one with the most satisfied constraints as the final prediction of the network. This is not necessarily the last assignment $\alpha^{\left(t_{\text{max}}^{\text{ev}}\right)}$.

**Input:** Instance (X,C), $t_{\text{max}}\in \mathbb{N}$.

**Output:** $\left(\varphi^{\left(1\right)},\ldots ,\varphi^{\left(t_{\text{max}}\right)}\right),\varphi^{\left(t\right)}:X\to {\left[0,1\right]}^{d}$.

**for** x∈X **do**.

//*random initialization*

$$s_{x}^{\left(0\right)}∼N{\left(0,1\right)}^{k}$$

$$h_{x}^{\left(0\right)}:=0\in {\mathbb{R}}^{k}$$

**for** $t\in \left\{1,\ldots ,t_{\text{max}}\right\}$ **do**.

**for** c:=(x,y,R)∈C **do**.

//*generate messages*

$$\left(m_{c,x}^{\left(t\right)},\;m_{c,y}^{\left(t\right)}\right):=S_{R}\left(s_{x}^{\left(t-1\right)},\;s_{y}^{\left(t-1\right)}\right)$$

**for** x∈X **do**.

//*combine messages and update*

$$r_{x}^{\left(t\right)}:=\frac{1}{\text{deg}\left(x\right)}\sum_{c\in C,x\in c}m_{c,x}^{\left(t\right)}$$

$$\left(h_{x}^{\left(t\right)},s_{x}^{\left(t\right)}\right):=\text{LSTM}\left(h_{x}^{\left(t-1\right)},\;s_{x}^{\left(t-1\right)},\;r_{x}^{\left(t\right)}\right)$$

$$\varphi^{\left(t\right)}\left(x\right):=\text{softmax}\left(W\cdot s_{x}^{\left(t\right)}\right)$$

**Algorithm 1:** Network Architecture.

**FIGURE 2:**
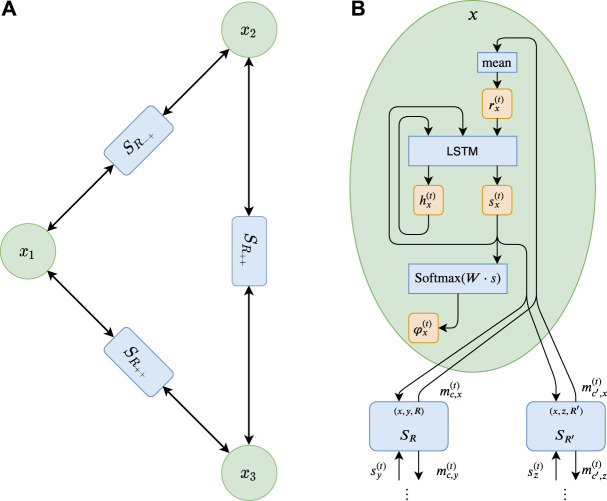
**(A)** The graph corresponding to the Max-2-SAT-instance f = (¬X_1_ $\vee x_{2})\wedge \left(x_{1}\vee x_{3}\right)\wedge \left(x_{2}\vee x_{3}\right)$. The nodes for the variables are shown in green. The functions through which the variables iteratively exchange messages are shown in blue **(B)** An illustration of the update mechanism of RUN-CSP. The trainable weights of this function are shared across all nodes, which allows RUN-CSP to process instances with arbitrary structure.

**Algorithm 1** specifies the architecture in pseudocode. Figure 2 illustrates the message passing graph for a Max-2-SAT instance and the internal update procedure of RUN-CSP. Note that the network’s output depends on the random initialization of the short-term states $s_{x}^{\left(0\right)}$. Those states are the basis for all messages sent during inference and thus for the solution found by RUN-CSP. By applying the network multiple times to the same input and choosing the best solution, we can therefore boost the performance.

We did evaluate more complex variants of this architecture with multi-layered messaging functions and multiple stacked recurrent cells. No increase in performance was observed with these modifications, while the running time increased. Replacing the LSTM cells with GRU cells slightly decreased the performance. Therefore, we use the simple LSTM-based architecture presented here.

### 3.2 Loss Function

In the following we derive our loss function used for unsupervised training. Let I=(X,D,C) be a CSP-instance. Assume without loss of generality that D={1,…,d} for a positive integer *d*. Given *I*, in every iteration our network will produce a soft variable assignment $\varphi :X\to {\left[0,1\right]}^{d}$, where φ(x) is stochastic for every x∈X. Instead of choosing the value with the maximum probability in φ(x), we could obtain a hard assignment α:X→D by independently sampling a value for each x∈X from the distribution specified by φ(x). In this case, the probability that any given constraint (x,y,R)∈C is satisfied by α can be expressed by

$$\underset{\alpha ∼\varphi }{\text{Pr}}\left[\left(\alpha \left(x\right),\alpha \left(y\right)\right)\in R\right]=\varphi {\left(x\right)}^{T}A_{R}\;\varphi \left(y\right)$$ (3)

where $A_{R}\in {\left\{0,1\right\}}^{d\times d}$ is the characteristic matrix of the relation *R* with ${\left(A_{R}\right)}_{i,j}=1\Leftrightarrow \left(i,j\right)\in R$. We then aim to minimize the combined negative log-likelihood over all constraints:

$${ℒ}_{\text{CSP}}\left(\varphi ,I\right):=\frac{1}{\left|C\right|}\cdot \underset{\left(x,y,R\right)\in C}{\sum }-\text{log}\left(\varphi {\left(x\right)}^{T}A_{R}\;\varphi \left(y\right)\right)$$ (4)

We combine the loss function ${ℒ}_{\text{CSP}}$ throughout all iterations with a discount factor λ∈[0,1] to get our training objective:

$$ℒ\left({\left\{\varphi_{t}\right\}}_{t\leq t_{\text{max}}^{\text{tr}}},I\right):=\overset{t_{\text{max}}^{\text{tr}}}{\underset{t=1}{\sum }}\lambda^{t_{\text{max}}^{\text{tr}}-t}\cdot {ℒ}_{\text{CSP}}\left(\varphi^{\left(t\right)},I\right)$$ (5)

This loss function allows us to train unsupervised since it does not depend on any ground truth assignments. Furthermore, it avoids reinforcement learning, which is computationally expensive. In general, computing optimal solutions for supervised training can easily turn out to be prohibitive; our approach completely avoids such computations.

We remark that it is also possible to extend the framework to weighted Max-CSPs where a real weight is associated with each constraint. To achieve this, we can replace the averages in the loss function and message collection steps by weighted averages. Negative constraint weights can be incorporated by swapping the relation with its complement. We demonstrate this in Section 4.2 where we evaluate RUN-CSP on the weighted Max-Cut problem.

## 4 Experiments

To validate our method empirically, we performed experiments for Max-2-SAT, Max-Cut, 3-COL and Max-IS. For all experiments, we used internal states of size k=128; state sizes up to k=1024 did not increase performance for the tested instances. We empirically chose to use $t_{\text{max}}^{\text{tr}}=30$ iterations during training and, unless stated otherwise, $t_{\text{max}}^{\text{ev}}=100$ for evaluation. Especially for larger instances it proved beneficial to use a relatively high $t_{\text{max}}^{\text{ev}}$. In contrast, choosing $t_{\text{max}}^{\text{tr}}$ too large during training ($t_{\text{max}}^{\text{tr}}>50$) resulted in unstable training. During evaluation, we use 64 parallel runs for each instance and use the best result. Further increasing this number mainly increases the runtime but has no real effect on the quality of solutions. We trained most models with 4,000 instances split into in 400 batches. Training is performed for 25 epochs using the Adam optimizer with default parameters and gradient clipping at a norm of 1.0. The decay over time in our loss function was set to λ=0.95. We provide a more detailed overview of our implementation and training configuration in the Supplementary Material.

We ran our experiments on machines with two Intel Xeon 8160 CPUs and one NVIDIA Tesla V100 GPU but got very similar runtime on consumer hardware. Evaluating 64 runs on an instance with 1,000 variables and 1,000 constraints takes about 1.5 s, 10,000 constraints about 5 s, and 20,000 constraints about 8 s. Training a model takes less than 30 min. Thus, the computational cost of RUN-CSP is relatively low.

### 4.1 Maximum 2-Satisfiability

We view Max-2-SAT as a binary CSP with domain D={0,1} and a constraint language consisting of three relations $R_{00}$ (for clauses with two negated literals), $R_{01}$ (one negated literal) and $R_{11}$ (no negated literals). For example, $R_{01}=\left\{\left(0,0\right),\left(0,1\right),\left(1,1\right)\right\}$ is the set of satisfying assignments for a clause (¬x∨y). For training a RUN-CSP model we used 4,000 random 2-CNF formulas with 100 variables each. The number of clauses was sampled uniformly between 100 and 600 for every formula and each clause was generated by sampling two distinct variables and then independently negating the literals with probability 0.5.

#### 4.1.1 Random Instances

For the evaluation of RUN-CSP in Max-2-SAT we start with random instances and compare it to a number of problem-specific heuristics. All baselines can solve Max-SAT for arbitrary arities, not only Max-2-SAT, while RUN-CSP can solve a variety of binary Max-CSPs. The state-of-the-art Max-SAT Solver *Loandra* (Berg et al., 2019) won the unweighted track for incomplete solvers in the Max-SAT Evaluation 2019 (Bacchus et al., 2019). We ran Loandra in its default configuration with a timeout of 20 min on each formula. To put this into context, on the largest evaluation instance used here (9,600 constraints) RUN-CSP takes less than 7 min on a single CPU core and about 5 s using the GPU. *WalkSAT* (Selman et al., 1993; Kautz, 2019) is a stochastic local search algorithm for approximating Max-Sat. We allowed WalkSAT to perform 10 million flips on each formula using its “noise” strategy with parameters n=2 and m=2000. Its performance was boosted similarly to RUN-CSP by performing 64 runs and selecting the best result.

**FIGURE 3:**
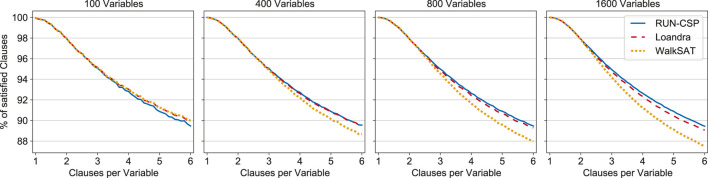
Percentage of satisfied clauses of random 2-CNF formulas for RUN-CSP, Loandra and WalkSAT. Each data point is the average of 100 formulas; the ratio of clauses per variable increases in steps of 0.1.

For evaluation we generated random formulas with 100, 400, 800, and 1,600 variables. The ratio between clauses and variables was varied in steps of 0.1 from 1 to 6. Figure 3 shows the average percentage of satisfied clauses in the solutions found by each method over 100 formulas for each size and density. The methods yield virtually identical results for formulas with less than 2 clauses per variable. For denser instances, RUN-CSP yields slightly worse results than both baselines when only 100 variables are present. However, RUN-CSP matches the results of Loandra for formulas with 400 variables and outperforms it for instances with 800 and 1,600 variables. The performance of WalkSAT degrades on these formulas and is significantly worse than RUN-CSP.

#### 4.1.2 Benchmark Instances

For more structured formulas, we use Max-2-SAT benchmark instances from the unweighted track of the Max-SAT Evaluation 2016 (Argelich, 2016) based on the Ising spin glass problem (De Simone et al., 1995; Heras et al., 2008). We used the same general setup as in the previous experiment but increased the timeout for Loandra to 60 min. In particular we use the same RUN-CSP model trained entirely on random formulas. Table 1 contains the achieved numbers of unsatisfied constraints across the benchmark instances. All methods produced optimal results on the first and the third instance. RUN-CSP slightly deviates from the optimum on the second instance. For the fourth instance RUN-CSP found an optimal solution while both WalkSAT and Loandra did not. On the largest benchmark formula, RUN-CSP again produced the best result.

**TABLE 1:** Max-2-SAT: Number of unsatisfied constraints for Max-2-SAT benchmark instances derived from the Ising spin glass problem.

Instance	|V|	|C|	Opt	RUN-CSP	WalkSAT	Loandra
t3pm3	27	162	17	17	17	17
t4pm3	64	384	38	40	38	38
t5pm3	125	750	78	78	78	78
t6pm3	216	1,269	136	136	142	142
t7pm3	343	2,058	209	216	227	225

Thus, RUN-CSP is competitive for random as well as spin-glass-based structured Max-2-SAT instances. Especially on larger instances it also outperforms conventional methods. Furthermore, training on random instances generalized well to the structured spin-glass instances.

### 4.2 Max Cut

Max-Cut is a classical Max-CSP with domain D={0,1} and only one relation $R_{\neq }=\left\{\left(0,1\right),\left(1,0\right)\right\}$ used in the constraints.

#### 4.2.1 Regular Graphs

In this section we evaluate RUN-CSP’s performance on this problem. Yao et al., (2019) proposed two unsupervised GNN architectures for Max-Cut. One was trained through policy gradient descent on a non-differentiable loss function while the other used a differentiable relaxation of this loss. They evaluated their architectures on random regular graphs, where the asymptotic Max-Cut optimum is known. We use their results as well as their baseline results for Extremal Optimization (EO) (Boettcher and Percus, 2001) and a classical approach based on semi-definite programming (SDP) (Goemans and Williamson, 1995) as baselines for RUN-CSP. To evaluate the sizes of graph cuts, Yao et al., (2019) introduced a relative performance measure called *P-value* given by $P\left(z\right)=\frac{z/n-d/4}{\sqrt{d/4}}$ where *z* is the predicted cut size for a *d*-regular graph with *n* nodes. Based on results of Dembo et al., (2017), they showed that the expected *P*-value of *d*-regular graphs approaches $P^{\text{*}}\approx 0.7632$ as n→∞. *P*-values close to $P^{\text{*}}$ indicate a cut where the size is close to the expected optimum and larger values are better. While Yao et al. trained one instance of their GNN for each tested degree, we trained one network model on 4,000 Erdős–Rényi graphs and applied it to all graphs. For training, each graph had a node count of n=100 and a uniformly sampled number of edges m∼U(100,2000). Thus, the model was not trained specifically for regular graphs. Table 2 reports the mean *P*-values across 1,000 random regular graphs with 500 nodes for different degrees. For every method other than RUN-CSP, we provide the values as reported by Yao et al. While RUN-CSP does not match the cut sizes produced by extremal optimization, it clearly outperforms both versions of the GNN as well as the classical SDP-based approach.

**TABLE 2:** Max-Cut: *P*-values of graph cuts produced by RUN-CSP, Yao, SDP, and EO for regular graphs with 500 nodes and varying degrees. We report the mean across 1,000 random graphs for each degree.

d	RUN-CSP	Yao Rel	Yao Pol	SDP	EO
3	0.714	0.707	0.693	0.702	0.727
5	0.726	0.701	0.668	0.690	0.737
10	0.710	0.670	0.599	0.682	0.735
15	0.697	0.607	0.629	0.678	0.736
20	0.685	0.614	0.626	0.674	0.732

#### 4.2.2 Benchmark Instances

We performed additional experiments on standard Max-Cut benchmark instances. The Gset dataset (Ye, 2003) is a set of 71 weighted and unweighted graphs that are commonly used for testing Max-Cut algorithms. The dataset contains three different types of random graphs. Those graphs are Erdős–Rényi graphs with uniform edge probability, graphs where the connectivity gradually decays from node 1 to *n*, and 4-regular toroidal graphs. Here, we use two unweighted graphs for each type from this dataset. We reused the RUN-CSP model from the previous experiment but increased the number of iterations for evaluation to $t_{\text{max}}^{\text{ev}}=500$. Our first baseline by Choi and Ye (2000) uses an SDP solver based on dual scaling (DSDP) and a reduction based on the approach of Goemans and Williamson (1995). Our second baseline Breakout Local Search (BLS) is based on the combination of local search and adaptive perturbation (Benlic and Hao, 2013). Its results are among the best known solutions for the Gset dataset. For DSDP and BLS we report the values as provided in the literature. Table 3 reports the achieved cut sizes for RUN-CSP, DSDP, and BLS. On G14 and G15, which are random graphs with decaying node degree, the graph cuts produced by RUN-CSP are similar in size to those reported for DSDP. For the Erdős–Rényi graphs G22 and G55 RUN-CSP performs better than DSDP but worse than BLS. Lastly, on the toroidal graphs G49 and G50 all three methods achieved the best known cut size. This reaffirms the observation that our architecture works particularly well for regular graphs. Although RUN-CSP did not outperform the state-of-the-art heuristic in this experiment it performed at least as well as the SDP based approach DSDP.

**TABLE 3:** Max-Cut: Achieved cut sizes on Gset instances for RUN-CSP, DSDP, and BLS.

Graph	|V|	|E|	RUN-CSP	DSDP	BLS
G14	800	4,694	2,943	2,922	3,064
G15	800	4,661	2,928	2,938	3,050
G22	2,000	19,990	13,028	12,960	13,359
G49	3,000	6,000	6,000	6,000	6,000
G50	3,000	6,000	5,880	5,880	5,880
G55	5,000	12,468	10,116	9,960	10,294

#### 4.2.3 Weighted Maximum Cut Problem

Additionally, we evaluate RUN-CSP on the weighted Max-Cut problem, where every edge e∈E has an associated weight $w_{e}\in \left\{1,-1\right\}$. The aim is to maximize the objective:

$$\text{Θ}\left(S,T\right)=\underset{e\in E\cap^{\text{​}}\left(S\times T\right)}{\sum }w_{e},$$

where the partition S,T of *V* defines a cut. We can apply RUN-CSP to this problem by training a model for the constraint language $\text{Γ}=\left\{R_{=},R_{\neq }\right\}$ over the domain D={0,1}. Here, $R_{=}$ and $R_{\neq }$ are the equality and inequality relations, respectively. We model every positive edge as a constraint with $R_{\neq }$ and every negative edge with $R_{=}$. We trained a RUN-CSP network on 4,000 random Erdős–Rényi graphs with n=100 nodes and m∼U(100,300) edges. The weights $w_{e}∼\left\{1,-1\right\}$ were drawn uniformly for each edge.

We evaluate this model on 10 benchmark instances obtained from the Optsicom Project^2^, namely the 10 smallest graphs of set 2. These instances are based on the lsing spin glass problem and are commonly used to evaluate heuristics empirically. All 10 graphs have n=125 nodes and m=375 edges. Khalil et al., (2017) utilize reinforcement learning to guide greedy search heuristics for combinatorial problems including weighted Max-Cut. They evaluated their method on the same benchmark instances for weighted Max-Cut and compared the performance to a classical greedy heuristic (Kleinberg and Tardos, 2006) and an SDP-based method (Goemans and Williamson, 1995). Furthermore, they approximated the optimal values by running CPLEX for 1 h on every instance. We use their reported results and baselines for a comparison with RUN-CSP. Crucially, Khalil et al., (2017) trained their network on random variations of the benchmark instances, while RUN-CSP was trained on purely random data. Table 4 provides the achieved cut sizes. On all but one benchmark instance RUN-CSP yields the largest cuts and on five out of 10 instances it even found the optimal cut value. The classical approaches based on Greedy Search and SDP performed substantially worse than both neural methods.

**TABLE 4:** Max-Cut: Achieved cut sizes on Optsicom Benchmarks. The optimal values were estimated by Khalil et al., (2017) by running CPLEX for 1 h on each instance.

Graphs	Opt	RUN-CSP	Khalil et al	Greedy	SDP
G54100	110	110	108	80	54
G54200	112	112	108	90	58
G54300	106	106	104	86	60
G54400	114	112	108	96	56
G54500	112	112	112	94	56
G54600	110	110	110	88	66
G54700	112	110	108	88	60
G54800	108	106	108	76	54
G54900	110	108	108	88	68
G541000	112	110	108	80	54
Approx. Ratio	1.0	1.01	1.02	1.28	1.90

### 4.3 Coloring

Within coloring we focus on the case of three colors, i.e., we consider CSPs over the domain {1,2,3} with the inequality relation $R_{\neq }$. In general, RUN-CSP aims to satisfy as many constraints as possible and therefore approximates Max-3-Col. Instead of evaluating on Max-3-Col, we evaluate on its practically more relevant decision variant 3-COL which asks whether a given graph is 3-colorable without conflicts. We turn RUN-CSP into a classifier by predicting that a given input graph is 3-colorable if and only if it is able to find a conflict-free vertex coloring.

#### 4.3.1 Hard Instances

We evaluate RUN-CSP on so-called “hard” random instances, similar to those defined by Lemos et al., (2019). These instances are a special subclass of Erdős–Rényi graphs where an additional edge can make the graph no longer 3-colorable. We describe our exact generation procedure in the Supplementary Material. We trained five RUN-CSP models on 4,000 hard 3-colorable instances with 100 nodes each. In Table 5 we present results for RUN-CSP, a greedy heuristic with DSatur strategy (Brélaz, 1979), and the state-of-the-art heuristic HybridEA (Galinier and Hao, 1999; Lewis et al., 2012; Lewis, 2015). HybridEA was allowed to make 500 million constraint checks on each graph. We observe that larger instances are harder for all tested methods and between the three algorithms there is a clear hierarchy. The state-of-the-art heuristic HybridEA clearly performs best and finds solutions even for some of the largest graphs. RUN-CSP finds optimal colorings for a large fraction of graphs with up to 100 nodes and even a few correct colorings for graphs of size 200. The weakest algorithm is DSatur which even fails on most of the small 50 node graphs and gets rapidly worse for larger instances.

**TABLE 5:** 3-COL: Percentages of hard 3-colorable instances for which optimal 3-colorings were found by RUN-CSP, Greedy, and HybridEA. We evaluate on 1,000 instances for each size. We provide mean and standard deviation across five different RUN-CSP models.

Nodes	RUN-CSP	Greedy	HybridEA
50	98.4 ± 0.3	34.0	100.0
100	62.5 ± 2.7	6.7	100.0
150	15.5 ± 2.3	1.5	98.7
200	2.6 ± 0.4	0.5	88.9
300	0.1 ± 0.0	0.0	39.9
400	0.0 ± 0.0	0.0	15.3

Choosing larger or more training graphs for RUN-CSP did not significantly improve its performance on larger hard graphs. We assume that a combination of increasing the state size, complexity of the message generation functions, and number and size of training instances is able to achieve better results, but on the cost of efficiency.

In Table 5 we do not report results for GNN-GCP by Lemos et al., (2019) as the structure of the output is fundamentally different. While the three algorithms in Table 5 output a coloring, GNN-GCP outputs a guess on the chromatic number without providing a proof that this is achievable. We trained instances of GNN-GCP on 32,000 pairs of hard graphs of size 40 to 60 (small) and 50 to 100 (medium). For testing, we restricted the model to only choose between the chromatic numbers 3 and 4, when allowing a wider range of possible values, the accuracy of GNN-GCP drops considerably. The network was able to achieve test accuracies of 75% (respectively 65% when trained and evaluated on medium instances). The model generalizes fairly well, with the small model achieving 64% on the medium test set and the large model achieving 74% on the small test set, almost matching the performance of the network trained on graphs of the respective size. On a set of test instances of hard graphs with 150 nodes, GNN-GCP achieved an accuracy of 52% (54% for the model trained on medium instances). Thus, the model performs significantly worse than RUN-CSP which achieves 81% (GNN-GCP 59%) accuracy on a test set of graphs of size 100, and 68% on graphs of size 150 where GNN-GCP achieves up to 54%. The numbers for RUN-CSP are larger than those reported in Table 5 since in the table only 3-colorable instances were considered. Here, the accuracy is computed over 3-colorable instances as well as their non-3-colorable counter parts. By design, RUN-CSP achieves perfect classification on negative instances.

Overall, we see that despite being designed for maximization tasks, RUN-CSP outperforms greedy heuristics and neural baselines on the decision variant of 3-COL for hard random instances.

#### 4.3.2 Structure Specific Performance

On the example of the coloring problem, we evaluate generalization to other graph classes. We expect a network trained on instances of a particular structure to adapt toward this class and outperform models trained on different graph classes. We briefly evaluate this hypothesis for four different classes of graphs.

**Erdős–Rényi Graphs:** Graphs are generated by uniformly sampling *m* distinct edges between *n* nodes.

**Geometric Graphs:** A graph is generated by first assigning random positions within a 1×1 square to *n* distinct nodes. Then an edge is added for every pair of points with a distance less than *r*.

**Powerlaw-Cluster Graphs:** This graph model was introduced by Holme and Kim (2002). Each graph is generated by iteratively adding *n* nodes and connected to *m* existing nodes. After each edge is added, a triangle is closed with probability *p*, i.e., an additional edge is added between the new node and a random neighbor of the other endpoint of the edge.

**Regular Graphs:** We consider random 5-regular graphs as an example for graphs with a very specific structure.

We trained five RUN-CSP models on 4,000 random instances of each type where each graph had between 50 and 100 nodes. We refer to these groups of models as $M_{\text{ER}}$, $M_{\text{Geo}}$, $M_{\text{Pow}}$ and $M_{\text{Reg}}$. Five additional models $M_{\text{Mix}}$ were trained on a mixed dataset with 1,000 random instances of each graph class. The exact parameters for generating the graphs can be found in the Supplementary Material. Note that the parameters for each class were purposefully chosen such that most graphs are not 3-colorable. This allows us to evaluate the relative performance on the maximization task. Table 6 contains the percentage of unsatisfied constraints over the models on 1,000 fresh graphs of each class. We observe that all models perform well on the class of structures they were trained on and $M_{\text{Reg}}$ yields the worst performance on all other classes. Both $M_{\text{Geo}}$ and $M_{\text{Pow}}$ outperform $M_{\text{ER}}$ on Erdős–Rényi graphs while $M_{\text{ER}}$ outperforms $M_{\text{Geo}}$ on Powerlaw-Cluster and $M_{\text{Pow}}$ on geometric graphs. When averaging over all four classes, $M_{\text{Mix}}$ produces the best results, despite not achieving the best results for any particular class. Additionally, we observe a very low variance in performance between the different models trained on the same dataset. Only the models trained on relatively narrow graph classes, namely regular graphs and to some extent also Powerlaw-Cluster graphs, exhibit a higher variance.

**TABLE 6:** Max-3-Col: Percentages of unsatisfied constraints for each graph class under the different RUN-CSP models. Values are averaged over 1,000 graphs and the standard deviation is computed with respect to the five RUN-CSP models.

Graphs	$M_{\text{ER}}$ (%)	$M_{\text{Geo}}$ (%)	$M_{\text{Pow}}$ (%)	$M_{\text{Reg}}$ (%)	$M_{\text{Mix}}$ (%)
Erdos-Renyi	4.75 ± 0.01	4.73 ± 0.02	4.72 ± 0.02	6.69 ± 1.60	4.73 ± 0.01
Geometric	10.33 ± 0.07	10.16 ± 0.04	11.39 ± 0.66	18.99 ± 3.32	10.18 ± 0.03
Pow. Cluster	1.89 ± 0.00	1.96 ± 0.01	1.87 ± 0.00	2.44 ± 0.67	1.89 ± 0.00
Regular	2.33 ± 0.01	2.41 ± 0.03	2.33 ± 0.02	2.32 ± 0.00	2.33 ± 0.00
Mean	4.83 ± 0.02	4.82 ± 0.03	5.08 ± 0.18	7.61 ± 1.40	4.78 ± 0.01

Overall, this demonstrates that training on locally diverse graphs (e.g., geometric graphs or a mixture of graph classes) leads to good generalization toward other graph classes. While all tested networks achieved competitive results on the structure that they were trained on, they were not always the best for that particular structure. Therefore, our original hypothesis appears to be overly simplistic and restricting the training data to the structure of the evaluation instances is not necessarily optimal.

### 4.4 Independent Set

Finally, we experimented with the maximum independent set problem Max-IS. The independence condition can be modeled through a constraint language ${\text{Γ}}_{\text{IS}}$ with one binary relation $R_{\text{IS}}=\left\{\left(0,0\right),\left(0,1\right),\left(1,0\right)\right\}$. Here, assigning the value 1 to a variable is interpreted as including the corresponding node in the independent set. Max-IS is *not* simply Max-CSP($\Gamma_{\text{IS}}$), since the empty set will trivially satisfy all constraints. Instead, Max-IS is the problem of finding an assignment which satisfies $R_{\text{IS}}$ at all edges while maximizing an additional objective function that measures the size of the independent set. To model this in our framework, we extend the loss function to reward assignments with many variables set to 1. For a graph G=(V,E) and a soft assignment φ:V→[0,1], we define

$$\begin{array}{c} {ℒ}_{\text{MIS}}\left(\varphi ,G\right)=\left(\kappa +{ℒ}_{\text{CSP}}\left(\varphi ,G\right)\right)\cdot \left(1+{ℒ}_{\text{size}}\left(\varphi ,G\right)\right),\\ {ℒ}_{\text{size}}\left(\varphi ,G\right)=\frac{1}{\left|V\right|}\underset{v\in V}{\sum }\left(1-\varphi \left(v\right)\right). \end{array}$$ (6)

Here, ${ℒ}_{\text{CSP}}$ is the standard RUN-CSP loss for ${\text{Γ}}_{\text{IS}}$ and κ adjusts the relative importance of ${ℒ}_{\text{CSP}}$ and ${ℒ}_{\text{size}}$. Intuitively, smaller values for κ decrease the importance of ${ℒ}_{\text{size}}$ which favors larger independent sets. A naive weighted sum of both terms turned out to be unstable during training and yielded poor results, whereas the product in Eq. 6 worked well. For training, ${ℒ}_{\text{MIS}}$ is combined across iterations with a discount factor λ as in the standard RUN-CSP architecture.

#### 4.4.1 Random Instances

**FIGURE 4:**
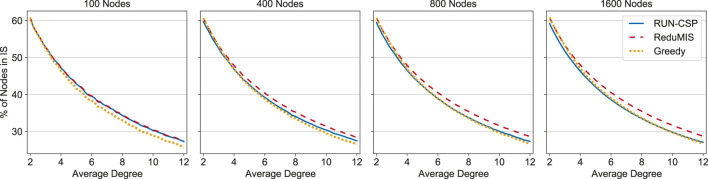
Independent set sizes on random graphs produced by RUN-CSP, ReduMIS and a greedy heuristic. The sizes are given as the percentage of nodes contained in the independent set. Every data point is the average for 100 graphs; the degree increases in steps of 0.2.

We start by evaluating the performance on random graphs. We trained a network on 4,000 random Erdős–Rényi graphs with 100 nodes and m∼U(100,600) edges each and with κ=1. For evaluation we use random graphs with 100, 400, 800 and 1,600 nodes and a varying number of edges. For roughly 6% of all predictions, the predicted set contained induced edges (just a single edge in most cases), meaning the predicted sets where not independent. We corrected these predictions by removing one of the endpoints of each induced edge from the set and only report results after this correction. We compare RUN-CSP against two baselines: ReduMIS, a state-of-the-art Max-IS solver (Akiba and Iwata, 2016; Lamm et al., 2017) and a greedy heuristic, which we implemented ourselves. The greedy procedure iteratively adds the node with lowest degree to the set and removes the node and its neighbors from the graph until the graph is empty. Figure 4 shows the achieved independent set sizes, each data point is the mean IS size across 100 random graphs. For graphs with 100 nodes, RUN-CSP achieves similar sizes as ReduMIS and clearly outperforms the greedy heuristic. On larger graphs our network produces smaller sets than ReduMIS. However, RUN-CSP’s performance remains similar to the greedy baseline and, especially on denser graphs, outperforms it.

#### 4.4.2 Benchmark Instances

For more structured instances, we use a set of benchmark graphs from a collection of hard instances for combinatorial problems (Xu, 2005). The instances are divided into five sets with five graphs each. These graphs were generated through the RB Model (Xu and Li, 2003; Xu et al., 2005), a model for generating hard CSP instances. A graph of the class frb*c*-*k* consists of *c* interconnected *k*-cliques and the MAX-IS has a forced size of *c*. The previous model trained on Erdős–Rényi graphs did not perform well on these instances and produced sets with many induced edges. Thus, we trained a new network on 2,000 instances we generated ourselves through the RB model. The exact generation procedure of this dataset is provided in the Supplementary Material. We set κ=0.1 to increase the importance of the independence condition. The predictions of the new model contained no induced edges for all benchmark instances. Table 7 contains the achieved IS sizes. We observe that RUN-CSP yields similar results to the greedy heuristic. While our network does not match the state-of-the-art heuristic, it beats the greedy approach on large instances with over 100,000 edges.

**TABLE 7:** Max-IS: Achieved IS sizes for the benchmark graphs. We report the mean and std. deviation for the five graphs in each group.

Graphs	|V|	|E|	RUN-CSP	Greedy	ReduMIS
frb30–15	450	18 k	25.8 ± 0.8	24.6 ± 0.5	30 ± 0.0
frb40–19	790	41 k	33.6 ± 0.5	33.0 ± 1.2	39.4 ± 0.5
frb50–23	1,150	80 k	42.2 ± 0.4	42.2 ± 0.8	48.8 ± 0.4
frb59–26	1,478	126 k	49.4 ± 0.5	48.0 ± 0.7	57.4 ± 0.9

## 5 Conclusions

We have presented a universal approach for approximating Max-CSPs with recurrent neural networks. Its key feature is the ability to train without supervision on any available data. Our experiments on the optimization problems Max-2-SAT, Max-Cut, 3-COL and Max-IS show that RUN-CSP produces high quality approximations for all four problems. Our network can compete with traditional approaches like greedy heuristics or semi-definite programming on random data as well as benchmark instances. For Max-2-SAT, RUN-CSP was able to outperform a state-of-the-art MAX-SAT Solver. Our approach also achieved better results than neural baselines, where those were available. RUN-CSP networks trained on small random instances generalize well to other instances with larger size and different structure. Our approach is very efficient and inference takes only a few seconds, even for larger instances with over 10,000 constraints. The runtime scales linearly in the number of constraints and our approach can fully utilize modern hardware, like GPUs.

Overall, RUN-CSP seems like a promising approach for approximating Max-CSPs with neural networks. The strong results are somewhat surprising, considering that our networks consist of just one LSTM cell and a few linear functions. We believe that our observations point toward a great potential of machine learning in combinatorial optimization.

### Future Work

We plan to extend RUN-CSP to CSPs of arbitrary arity and to weighted CSPs. It will be interesting to see, for example, how it performs on 3-SAT and its maximization variant. Another possible future extension could combine RUN-CSP with traditional local search methods, similar to the approach by Li et al., (2018) for Max-IS. The soft assignments can be used to guide a tree search and the randomness can be exploited to generate a large pool of initial solutions for traditional refinement methods.

## Data Availability

The code for RUN-CSP including the generated datasets and their generators can be found on github https://github.com/toenshoff/RUN-CSP. The additional datasets can be downloaded at their sources as specified in the following: Spinglass 2-CNF (Heras et al., 2008), http://maxsat.ia.udl.cat/benchmarks/ (Unweighted Crafted Benchmarks); Gset (Ye, 2003), https://www.cise.ufl.edu/research/sparse/matrices/Gset/; Max-IS Graphs (Xu, 2005), http://sites.nlsde.buaa.edu.cn/kexu/benchmarks/graph-benchmarks.htm; Optsicom (Corberán et al., 2006), http://grafo.etsii.urjc.es/optsicom/maxcut/.

## Footnotes

1 Our Tensorflow implementation of RUN-CSP is available at https://github.com/toenshoff/RUN-CSP.

2
http://grafo.etsii.urjc.es/optsicom/maxcut/.

## Acronyms

**RUN-CSP** Recurrent Unsupervised Neural Network for Constraint Satisfaction Problems.

**3-COL** 3-Coloring Problem.

**CSP** Constrain Satisfaction Problem.

**GNN** Graph Neural Network.

**MVC** Maximum Vertex Cover Problem.

**M** **ax** **-C** **ut** Maximum Cut Problem.

**M** **ax** **-2-S** **AT** Maximum Satisfiability Problem for Boolean formulas with two literals per clause.

**M** **ax** **-3-C** **ol** Maximum 3-Coloring Problem.

**M** **ax** **-IS** Maximum Independent Set.

**TSP** Traveling Sales Person Problem.
