# Supplementary material for: Graph Neural Networks for Maximum Constraint Satisfaction
Source: Front Artif Intell. 2021 Feb 25;3:580607. doi: 10.3389/frai.2020.580607 (PMC7959828; doi:10.3389/frai.2020.580607)
Supplement: Supplementary file 1 [file datasheet1.pdf]

Supplementary Material

1 REPRODUCIBILITY

This section summarizes the necessary information for replicating our implementation and exact training procedure. All trainable matrices in the messaging functions S_R were initialized with uniform Glorot initialization. For activation, bias, and initialization of the LSTM cell we used the default values provided by TensorFlow 1.15.0. All trainable parameters were regularized with the ℓ^2-norm with a weight of 0.01. The pooled vectors of received messages $r_x^{(t)}$ were normalized with an additional batch normalization layer, before being passed into the LSTM cell. The discount factor in our loss function was set to $\lambda = 0.95$ in all experiments. The state size of all networks was set to $k = 128$.

All networks were trained with $t_{\text{max}}^{\text{tr}} = 30$. Training was performed with the Adam optimizer using the default parameters $\beta_1 = 0.9$, $\beta_2 = 0.999$, and $\epsilon = 1 \times 10^{-7}$. The learning rate was initialized as 0.001 and decayed with a factor of 0.1 every 5 epochs. The gradients were clipped at a norm of 1.0.

1.1 Boosting

We perform 64 runs on each instance to boost the performance during evaluation. Our implementation performs these runs in parallel with a single forward pass of the network. To achieve this, we copy the graph 64 times and combine the disjoint copies into one larger instance. This instance is then processed by the RUN-CSP network. Since there are no messages exchanged between connected components, this is equivalent to executing the network 64 times on the same graph. Evaluating on a single large graph is, especially for small instances, faster on a GPU than multiple executions on the small graphs.

For the largest MAX-IS benchmark graphs the parallel inference with 64 copies exceeds the memory limit of our GPU. Instead, we performed 8 consecutive runs with 8 copies each and selected the best assignment afterwards.

2 GENERATED DATA

2.1 Hard 3-Col Instances

Here, we describe how the 'hard' 3-col graphs used in our vertex coloring experiment are generated. These instances are 3-colorable Erdős–Rényi graphs and there is (at least) one edge e such that when adding e to the graph, it is non-3-colorable. To generate such a graph, we first initialize a graph with n nodes and no edges. We then iteratively add individual edges which are uniformly sampled at random. After adding each edge, we use a conventional SAT solver to check whether the graph is still 3-colorable. (We use the pycosat Python package for this task.) This process is stopped once the graph is found to be non-3-colorable. The graphs with and without the last added edge are then returned as negative and positive instances, respectively. Our graph generation procedure stops adding edges when the next randomly chosen edge makes the graph non-3-colorable. In contrast, Lemos et al. (2019) stopped adding edges when it was possible to add such an edge that makes the graph non-3-colorable. The graphs generated by Lemos et al. are thus less dense than ours.

2.2 Structure Specific Performance for 3-COL

We compared the relative performance of RUN-CSP models when training and testing on Erdős–Rényi, Powerlaw-Cluster, geometric and regular graphs. Here, we provide the exact parameters used to generate the graphs in each structure class. The number of nodes was sampled uniformly between 50 and 100 for each graph of all four classes. For Erdős–Rényi graphs, the edge count m was chosen randomly between

100 and 400. The parameter r of each geometric graph was sampled uniformly from the interval $[0.1, 0.2]$. For Powerlaw-Cluster graphs, the parameter m was uniformly sampled from $\{1, 2, 3\}$ and p was uniformly drawn from the interval $[0, 1]$.

2.3 MAX-IS Benchmarks

The RUN-CSP network that was evaluated on the MAX-IS benchmark graphs was trained on our own synthetic benchmark instances. Here, we will describe the generation procedure of these instances. Xu and Li (2003); Xu et al. (2005) proposed the RB Model, which is a general model for generating hard random CSP instances close to the phase change of satisfiability. Furthermore, they described how to generate hard instances for graph problems, including MAX-IS instances, by reducing SAT benchmarks of the RB Model to these problems. We used their generation procedure for MAX-IS benchmarks as described in Xu (2005). Given $c \in \mathbb{N}$, $p \in [0, 1]$ and $\alpha, r > 0$, the procedure generates a graph as follows:

1. Generate c disjoint cliques with $k = c^{\alpha}$ vertices each.
2. Select two random cliques and generate $pc^{2\alpha}$ random edges between them (without repetition).
3. Run Step 2 for another $rc \ln c - 1$ times (with repetition).

To enforce an optimal independent set of size c, one can exclude one node of each clique from the process of adding random edges.

We used this procedure to generate 2,000 training instances. For each graph we uniformly sampled $c \sim U(10, 25)$, $k \sim U(5, 20)$ and $p \sim U(0.3, 1.0)$. We then chose

$$\alpha = \frac{\ln(k)}{\ln(c)} \quad \text{and} \quad r = -\frac{\alpha}{\ln(1-p)} \tag{S1}$$

This choice for r is expected to yield 'hard' instances according to the RB Model Xu et al. (2005). We then used the algorithm described above to generate a graph with the chosen parameters.

Training was performed with a batch size of 5. Note that we reduced the batch size in comparison to all other experiments due to the relatively large size of the graphs. The constant κ that distributes the importance of the losses $\mathcal{L}_{\text{CSP}}$ and $\mathcal{L}_{\text{size}}$ was reduced to $\kappa = 0.1$ to emphasize the independence condition. Without this reduction the computed solutions contained multiple edges violating independence and not just one or two as in the other IS experiment.

3 ADDITIONAL EXPERIMENTS

3.1 Coloring Benchmark Instances

We evaluated RUN-CSP on a number of k-COL benchmark instances, similar to Lemos et al. (2019). We obtained the 20 graphs from the COLOR02 Workshop[1] that were also used to evaluate GNN-GCP to enable a direct comparison. Any single RUN-CSP network is bound to a fixed domain size d. A network cannot use more than d colors and even if a given graph can be colored with less than d colors, RUN-CSP will still use all d colors. Thus, in order to compute a chromatic number, we trained 14 distinct networks with domain sizes ranging from 4 to 17. We apply each network to a given graph and choose the output that achieved a conflict free coloring with the fewest colors as our final result.

Unlike our experiments for MAX-CUT, we found that RUN-CSP networks trained purely in Erdős–Rényi graphs performed poorly in the given setup. Instead, we generated mixed datasets that consist of 30%

[1] The graphs can be downloaded at `https://mat.tepper.cmu.edu/COLOR02/`

Table S1. Results for k-COL on benchmarks instances. We provide the number of colors needed for an optimal coloring by RUN-CSP, Greedy and HybridEA. For GNN-GCP we provide the prediction as reported by Lemos et al. (2019). This method only predicts the chromatic number and can therefore underestimate the true value.

Benchmark	$\|V\|$	Opt	RUN-CSP	GNN-GCP	DSatur	HybridEA
Queen5_5	25	5	**5**	6	**5**	**5**
Queen6_6	36	7	8	**7**	8	**7**
myciel5	47	6	**6**	5	**6**	**6**
Queen7_7	49	7	10	8	9	**7**
Queen8_8	64	9	11	8	10	**9**
1-Insertions_4	67	4	5	**4**	5	5
huck	74	11	**11**	8	**11**	**11**
jean	80	10	**10**	7	**10**	**10**
Queen9_9	81	10	17	9	12	**10**
david	87	11	**11**	9	**11**	**11**
Mug88_1	88	4	**4**	3	**4**	**4**
myciel6	95	7	8	**7**	**7**	**7**
Queen8_12	96	12	17	10	13	**12**
games120	120	9	**9**	6	**9**	**9**
Queen11_11	121	11	> 17	12	15	12
anna	138	11	**11**	**11**	**11**	**11**
2-Insertions4	149	4	5	**4**	5	5
Queen13_13	169	13	> 17	14	17	14
myciel7	191	8	9	NA	**8**	**8**
homer	561	13	17	14	**13**	**13**

Erdős–Rényi graphs, 30% Geometric graphs, 30% Powerlaw-Cluster graphs and 10% Connected Caveman graphs Watts (1999). Connected Caveman graphs are a graph model introduced by Watts (1999) that depends on two numbers $l, k \in \mathbb{N}$. All other graph classes were introduced in the Section 5.2.

The training datasets were adapted to the number $c \in \{4, \dots, 17\}$ of colors available such that networks with more colors were trained on denser graphs. Each training set contained 4,000 random graphs generated according to the following parameters:

Erdős–Rényi: $n \sim U(50, 100)$, $m \sim U(2n, n \cdot c)$
Geometric: $n \sim U(80, 28c)$, $r \sim U(0.1, 0.2)$
Powerlaw-Cluster: $n \sim U(20, 20c)$, $m \sim U(1, 4)$, $r \sim U(1, 2)$
Connected Caveman: $l \sim U(10, 20)$, $k \sim U(\max(4, c-2), c+2)$

We compare RUN-CSP to the classical methods used in our previous vertex coloring experiment, namely HybridEA and the greedy DSatur strategy. As before, HybridEA was allowed to perform 500 million constraint checks on each graph. Table S1 provides the number of colors that each method needed to color the graphs without conflict. For comparison, we provide the predicted chromatic number of GNN-GCP as reported by Lemos et al. (2019). On most benchmark instances HybridEA finds the optimal chromatic number and otherwise uses one additional color. For instances with a chromatic number of up to 6 the performance of our network is identical to DSatur and HybridEA. In general, RUN-CSP performs slightly worse than the greedy DSatur algorithm. The number of instances for which RUN-CSP found optimal solutions is larger than the number of graphs for which GNN-GCP predicted the correct chromatic number. We point out that the focus of our architecture is a maximization task associated with a domain of fixed size. Despite this, RUN-CSP was able to outperform GNN-GCP on this task, while also predicting color assignments.

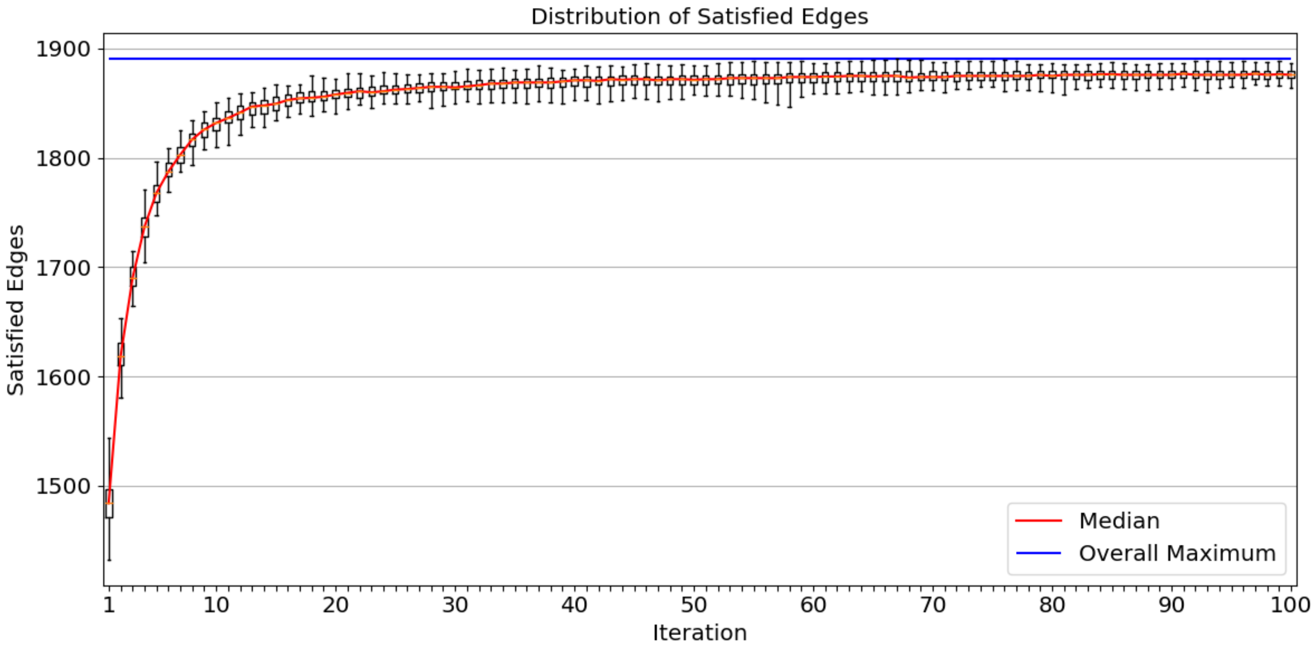

Figure S1. The distribution of satisfied edges for 64 parallel evaluation runs on a random Erdős–Rényi graph. For each iteration we show a boxplot that represents the 64 numbers of satisfied edges at a given time step. The whiskers of the boxplots include the minimum and maximum values. The limits of the box are given by the upper and lower quartile. The red line represents the median in each iteration. The overall maximum (reached after 67 steps) is shown as a horizontal blue line.

3.2 Convergence of RUN-CSP

We now use the MAX-3-COL problem to illustrate the convergence behavior of RUN-CSP networks. We generated a random Erdős–Rényi graph with 500 nodes and 2,000 edges. One of the RUN-CSP models of M_{Mix} from the previous experiment was used to predict a maximum 3-coloring for this graph. We performed 64 parallel evaluation runs for $t^{\text{ev}}_{\text{max}} = 100$ iterations. For each iteration we obtain 64 values for the numbers of correctly colored edges, one for each parallel run. Figure S1 plots the distributions of these values across all iterations as boxplots. In the first 20 time steps, the predictions improve quickly, as the network moves away from its random initialization towards a better solution. The network continues to find better color assignments far past iteration 30, which is the number iterations used during training, the absolute maximum was reached in iteration 67.

REFERENCES

Lemos, H., Prates, M., Avelar, P., and Lamb, L. (2019). Graph colouring meets deep learning: Effective graph neural network models for combinatorial problems. *arXiv preprint arXiv:1903.04598*

Watts, D. J. (1999). Networks, dynamics, and the small-world phenomenon. *American Journal of sociology* 105, 493–527

[Dataset] Xu, K. (2005). BHOSLIB: Benchmarks with hidden optimum solutions for graph problems (maximum clique, maximum independent set, minimum vertex cover and vertex coloring)

Xu, K., Boussemart, F., Hemery, F., and Lecoutre, C. (2005). A simple model to generate hard satisfiable instances. In *IJCAI-05, Proceedings of the Nineteenth International Joint Conference on Artificial Intelligence, Edinburgh, Scotland, UK, July 30 - August 5, 2005*. 337–342

Xu, K. and Li, W. (2003). Many hard examples in exact phase transitions with application to generating hard satisfiable instances. *arXiv preprint cs/0302001*
